# Modelling human diabetes *ex vivo*: a glance at maturity onset diabetes of the young

**DOI:** 10.3389/fendo.2024.1427413

**Published:** 2024-09-25

**Authors:** Moustapha Ka, Eleanor Hawkins, Celio Pouponnot, Bertrand Duvillié

**Affiliations:** ^1^ Department of Signaling, Radiobiology and Cancer, Institut Curie, Orsay, France; ^2^ INSERM U1021, Centre Universitaire, Orsay, France; ^3^ CNRS UMR 3347, Centre Universitaire, Orsay, France; ^4^ Université Paris-Saclay, Orsay, France; ^5^ PSL Research University, Paris, France; ^6^ Equipe Labellisée par la Ligue contre le cancer, Orsay, France

**Keywords:** stem cells, organoids, pancreas, islets, diabetes, MODY

## Abstract

Diabetes is a complex metabolic disease which most commonly has a polygenic origin; however, in rare cases, diabetes may be monogenic. This is indeed the case in both Maturity Onset Diabetes of the Young (MODY) and neonatal diabetes. These disease subtypes are believed to be simpler than Type 1 (T1D) and Type 2 Diabetes (T2D), which allows for more precise modelling. During the three last decades, many studies have focused on rodent models. These investigations provided a wealth of knowledge on both pancreas development and beta cell function. In particular, they allowed the establishment of a hierarchy of the transcription factors and highlighted the role of microenvironmental factors in the control of progenitor cell proliferation and differentiation. Transgenic mice also offered the possibility to decipher the mechanisms that define the functional identity of the pancreatic beta cells. Despite such interest in transgenic mice, recent data have also indicated that important differences exist between mice and human. To overcome these limitations, new human models are necessary. In the present review, we describe these *ex vivo* models, which are created using stem cells and organoids, and represent an important step toward islet cell therapy and drug discovery.

## Introduction

Diabetes is a complex metabolic disease characterized by chronic hyperglycemia and categorized into two types: insulino-dependent (Type 1, T1D) and non-insulino-dependent diabetes (Type 2, T2D). T1D has a well-established auto-immune origin, while T2D is associated with insulin-resistance and overweight status. However, in the current context, this classification appears to be oversimplified due to the overlap between symptoms, and a sub-classification may be required. In 2018, Ahlqvist et al. performed a data-driven cluster analysis based on 6 parameters: glutamate decarboxylase antibodies, age at diagnosis, body mass index (BMI), Hba1c, beta-cell function, and insulin-resistance. This study identified 5 clusters of patients with diabetes, with distinct characteristics which may be associated with potential complications (1). T1D and T2D have a polygenic origin, making identification of the etiology of onset and progression of diabetes complicated. Conversely, some specific forms of diabetes, including maturity onset diabetes of the young (MODY) and neonatal diabetes, are monogenic. Analysis of these particular diseases may help to improve our understanding of the molecular events underlying pancreatic beta-cell dysfunction and the initiation of diabetes. Moreover, it should be noted that in recent decades, most studies in this field were performed using rodent models. One important advantage of rodent models is the ease of genetic manipulation, which allows the performance of metabolic analyses at the whole organism level. However, recent research has highlighted several important differences between mice and human. One example is the inter-species difference in the two transcription factors of the GATA family, GATA 4 and GATA 6. In humans, heterozygous mutations in GATA 6 lead to pancreas agenesis and neonatal diabetes (2, 3), while GATA 4 haploinsufficiency causes neonatal and childhood-onset diabetes (4, 5). Conversely, in mice, single mutations of GATA 4 or GATA 6 have no impact on pancreas development or glucose homeostasis (6, 7); however, co-existing mutations on three of the four alleles of GATA4/6 produce a phenotype similar to that observed in humans. These observations indicate a functional redundancy between GATA4 and GATA6 in mice that does not exist in human. Thus, such inter-species differences need to be considered not only for delineating the human-specific molecular pathways that contribute to the disease, but also when choosing the most appropriate treatment for patients. Recently, significant progress has been made in the generation of new *ex vivo* models to study human diabetes. These models aim to reproduce the physiological development of beta cells, their interaction with the microenvironment, and their biology. In the present review, we will describe how these innovative approaches can be used in research to help better understand and treat monogenic forms of diabetes.

## Monogenic forms of diabetes

**Figure 1:**
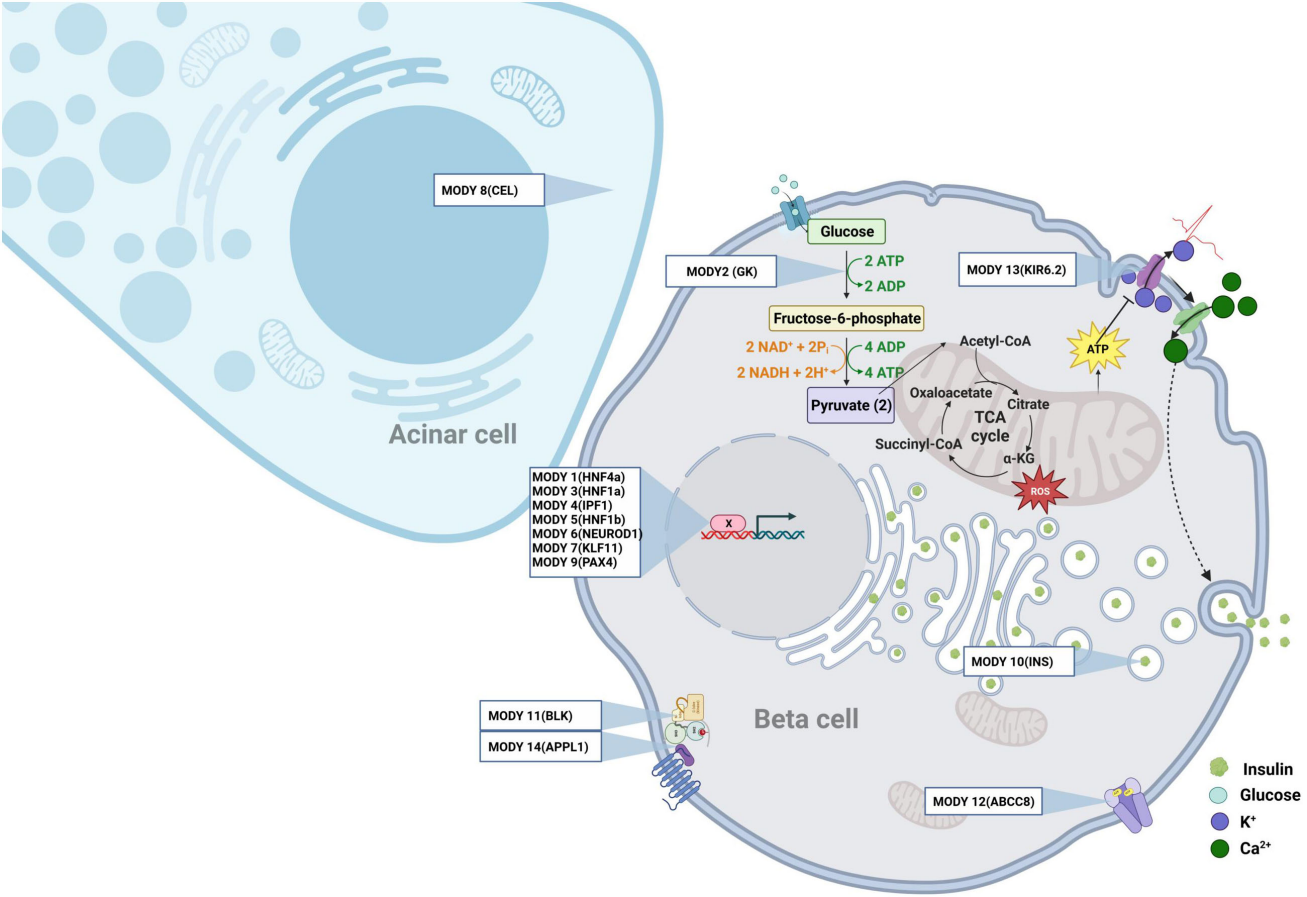
The monogenic diabetes. Representation at the cellular level of the different genes involved in Mody.

Monogenic diabetes, also known as maturity onset diabetes of the young (MODY), is a clinically heterogenous disease characterized by nonketotic diabetes mellitus and defects in pancreatic beta cell function, with an autosomal dominant inheritance pattern. MODY generally develops before the age of 25 years, and is frequent during childhood and adolescence. MODY represents 3-5% of all cases of diabetes. Interestingly, some MODY genes have been also associated with T1D and T2D (8–11), indicating an overlap between the different types of diabetes. Thus far, causative mutations in at least 14 genes have been characterized. However, the fact that causative mutations remain unidentified in 15-20% of families with MODY (French AJD Association, “Aide aux Jeunes Diabétiques”) indicates that other MODY associated genes remains to be discovered. The known causative genes of MODY (**Figure 1**) include hepatocyte nuclear factor (HNF) 4a [MODY 1 (12)], the glycolytic enzyme glucokinase [GK, MODY2 (13, 14)], HNF1a [MODY3 (15)], insulin promoting factor 1 [IPF1, MODY 4 (16)], HNF1b [MODY 5 (17)], neurogenic differentiation factor 1 also named BETA2 [MODY 6 (18)], KLF transcription factor 11 [KLF11, MODY 7 (19, 20)], carboxyl ester lipase [CEL, MODY 8 (21)], the transcription factor Paired box 4 [PAX4, MODY9 (22)], Insulin [MODY10 (23)], the tyrosine protein kinase BLK [MODY11 (24)], the ATP binding cassette subfamily C member 8 ABCC8 [MODY 12 (25)], the Potassium Inwardly Rectifying Channel Subfamily J member 11 KCNJ11, which encodes for the Kir6.2 subunit of the ATP-sensitive potassium channel in the pancreatic beta cell [MODY 13 (26)], the Adaptor Protein Phosphotyrosine interacting with PH domain and Leucin Zipper 1 APPL1 [MODY14 (27)]. Further research has shown that neonatal diabetes and syndrome-associated diabetes are also caused by single gene mutations (28). These forms are commonly underdiagnosed, and require better characterization. A recently-discovered new candidate gene for MODY is v-Maf avian musculoaponeurotic fibrosarcoma oncogene homolog A (MAF-A). MAF-A is a transcription factor that controls glucose stimulated insulin gene expression (GSIS) and insulin secretion (29). Prior research using MafA knock-out mice has revealed alterations in GSIS and disruption of the architecture of the pancreatic islets (30). Recently, two different missense mutations in human MAFA, at p.Ser64Phe and p.Thr57Arg, were detected in three unrelated families (31, 32). Intriguingly, both mutations caused both insulinomatosis (predominantly in females) and a MODY-like diabetes mellitus (predominantly in males). Iacovazzo et al. also showed that the p.Ser64Phe mutation impaired the phosphorylation of MAF-A by GSK3 resulting in an enhanced transactivation activity and increased MAF-A protein stability according to our previous work (33). It is probable that the p.Thr57Arg mutation will have a similar effect, as this mutation affects one of the residues phosphorylated by GSK3 (34, 35).

## Modelling monogenic diabetes using stem-cell derived beta cells

Recently, several new models have been developed to study MODY diabetes (**Table 1**). In particular, a variety of models can be used to study the biology of human beta cells, among which immortalized cells are commonly used. Several such cell lines have been produced and well characterized. For example, Blanchi et al. produced EndoC-bH5 human beta cells that show robust and highly-reproducible insulin secretion in response to glucose stimulation (36). These cells were initially produced by the integrative gene transfer of the immortalization genes hTERT and large T antigen to amplify the cells. Next, the transgenes were removed using a Cre recombinase system to facilitate physiological studies. However, this model cannot be used to investigate the process of differentiation. Adult pancreatic islets from cadaveric donors also represent a useful model, but these are scarce, difficult to genetically manipulate, and cannot be used to model beta cell development.

**Table 1:** The new human models for the study of MODY Diabetes.

Type	Application	Ref
EndoCbH5 Human beta-cells	Analysis of insulin secretion, metabolic activity, and drug screening	(36)
Induced Pluripotent Stem Cells derived beta-cells	Investigation of the effects of mutations in beta-cell differentiation and physiology. Drug Screening. Beta-cells replacement trials.	(37–54)
Pancreatic organoids	Study of the roles of genetic factors and microenvironment. Drug discovery.	(55–60)
Microfluidic Multi-organoids System	Study of multi-organ interaction and drug discovery	(61)

More recently, the landscape of diabetes models has significantly progressed thanks to the introduction of human pluripotent stem cells (hPSC), including embryonic stem cells (ESCs) and induced pluripotent stem cells (iPSCs). By studying the signals that govern the multiple steps of beta cell development *in vivo*, several protocols to generate insulin-secreting cells from iPS cells have been constantly improved. The first trials using hPSCs succeeded in generating pancreatic endoderms (62). Subsequently, pancreatic progenitor cells were obtained *in vitro*; however, transplantation into recipient mice was required to achieve complete endocrine differentiation (63). Finally, islet beta-like cells were produced from hPSCs *in vitro*, and were subsequently validated to reverse diabetes in mice (64, 65). Discoveries such as these have greatly increased interest in the field of cell therapy for diabetes. More recently, a number of studies have optimized the efficiency of terminal differentiation of beta cells, allowing more accurate investigation of their physiology (37–39). At present, research using stem cell islet technology has revealed several key features of human pancreas development and diabetes. Further, research combining disease modeling with gene editing and next generation sequencing has revealed the effects of mutations related to diabetes on multiple islet cell types (40) (see the modelization of monogenic diabetes using iPS cells in **Table 2**).

**Table 2:** Modelisation of monogenic diabetes using iPS cells.

Type	Gene	Cell origin	Characteristics	Phenotype	Ref
MODY1	HNF4a	iPS from fibroblasts	nonsense mutation (**p.Gln268Ter**) in exon 7 and impaired dimerization	elevated insulin, glucagon and somatostatin gene expression	(44)
			**p.Ile271AsnfsX3** mutation inducing loss of fonction	Impaired development due to down regulation of pancreatic transcriptions factors	(46)
MODY2	GK	iPS from Blood cell	**p.Leu146Pro** (c.437T>C) mutation	to be investigated	(47)
MODY3	HNF1a	iPS from fibroblasts	**p.His126Asp** mutation	deregulation of HNF1a target genes with GLUT2 downregulation	(48)
MODY4	PDX1	iPS from fibroblasts	**p.Pro33Thr** or **p.Cys18Arg** mutation	reduction of pancreatic progenitors, downregulation of transcriptions factors and reduced insulin synthesis and secretion	(49, 50)
MODY5	HNF1b	iPS from fibroblasts	premature termination codon in exon 2 **p.Arg177Ter** mutation	Decreased expression of HNF1b	(52)
MODY8	CEL	iPS from fibroblasts	**p.Pro606fsX100** (c.1818delC)	differentiation into endocrine cells, and normal β cell function	(53)

Mutations responsible for MODY are written in bold.

MODY1 patients with HNF4α mutations have been shown to exhibit some alterations in beta cell function, but with normal insulin sensitivity (41, 42), concurrent with impairments in the secretion of glucagon and pancreatic polypeptide (41, 43). Treatments for MODY1 generally aim to increase insulin levels, while hypoglycemic drugs such as sulfonylureas can also be used (44). To establish a MODY 1 model, Braverman-Gross et al. used fibroblasts from two patients harboring a nonsense mutation in exon 7 of HNF4a (p.Gln268Ter) (44). This mutation impairs the dimerization of HNF4a and its transactivation domain. The authors subsequently analyzed the genetic signature of the pancreatic progenitors following induction of differentiation, revealing an increase in expression of stage specific transcription factors, as well as elevated expression of the insulin, glucagon, and somatostatin genes in MODY1 cells. This observation reflects the features of hyperinsulinemia observed in neonatal MODY1 patients (45), and may also correspond to a compensatory mechanism that favors pancreatic development in the presence of HNF4a heterozygosity. The differentiation of the primitive gut tube was also analyzed, revealing an enrichment of clusters for apolipoproteins, triglyceride catabolic process, lipoprotein metabolic process, lipid metabolic process, hormone biosynthesis and secretion in MODY 1 cells. This observation could be related to the dyslipidemia observed in MODY 1 patients. Moreover, NG et al. generated hepatopancreatic forgut endoderm cells, in addition to hepatic and beta-like cells, using hiPS cells carrying the p.Ile271AnsfsX3 mutation extracted from a MODY1 family (46). These authors showed that HNF4a haploinsufficiency alters foregut development, as well as both hepatic and pancreatic cell fates. Hepatic and beta-cell gene signatures were also impaired. This study therefore indicates that, in this model, foregut abnormalities further extend to the liver and the pancreas. For MODY2, a human iPS cell line, QBRli010-A, with a mutation in the GCK gene (pLeu146Pro, c.437T>C) was generated (47). This iPS cell line displays pluripotency characteristics and is able to produce the three germ layers. However, further studies will be necessary to generate beta cells and model diabetes in relation to MODY2.

Another study investigated MODY3, caused by mutation in HNF1a. Indeed, this disease is characterized by an alteration in insulin secretion; however, the specific molecular mechanisms in humans remain unclear. Su Jun Low et al. derived iPS cells carrying a p.His126Asp mutation in HNF1a from a MODY3 patient. Genome wide RNASeq and Chip Seq analysis on hiPS-derived endocrine progenitors showed that many HNF1a target genes were deregulated. Importantly, they also found a strong decrease in the expression of the glucose transporter, GLUT2, resulting in reduced glucose uptake and ATP production in MODY 3 hIPS derived beta-cells. Thus, these data demonstrate the role of HNF1a in the regulation of GLUT2 as well as several genes that regulate insulin secretion (48).

Pancreatic agenesis is caused by a homozygous mutation in the homeobox gene *PDX1* (IPF1), while heterozygous mutations lead to MODY4 or T2D. Two iPS cell lines have previously been generated by episomal reprogramming of cells extracted from patients with missense coding mutations in the *PDX1* gene. The first patient was a woman with a p.Cys18Arg mutation in *PDX1* (49), and the second was a woman carrying a p.Pro33Thr mutation in the transactivation domain of *PDX1* (50). These cell lines represent useful tools to delineate the molecular events that precede MODY4 (51). Isogenic cell lines carrying homozygous PDX1^p.Cys18Arg/p.Cys18Arg^ and PDX1^p.Pro33Thr/p.Pro33Thr^ mutations were also generated. Interestingly, the heterozygous PDX1^p.Pro33Thr/+^, PDX1^p.Cys18Arg/+^, and homozygous PDX1^p.Pro33Thr/p.Pro33Thr^ and PDX1^p.Cys18Arg/p.Cys18Arg^ mutations were found to alter beta-cell differentiation and function, while the PDX1^p.Pro33Thr/p.Pro33Thr^ mutation also reduced the differentiation of pancreatic progenitors. This event is caused by the down-regulation of PDX1-bound genes. Together, these results demonstrate that all these mutations affect the endocrine lineages and participate in the development of diabetes.

Yabe et al. also analyzed MODY 5 using iPS cells from a Japanese patient to generate pancreatic beta cells (52). The iPS derived beta cells carried a MODY 5 mutation p.Arg177Ter, leading to a premature termination codon in exon 2 of HNF1b. The authors showed that the p.Arg177Ter mutant transcripts showed decreased expression compared to the wild type transcripts. They thus hypothesized that the mutant mRNA may be degraded by the nonsense-mediated decay pathway (NMD). Using cycloheximide to inhibit NMD, treatment increased the sequence signal of p.Arg177Ter mutant mRNA as compared to the controls, thus confirming their hypothesis.

More recently, Pelligrini et al. found a heterozygous pathogenic variant (p.Pro606fsX100, c.1818delC) in the CEL gene encoding carboxyl ester lipase (MODY8) (53). CEL is expressed in pancreatic acinar cells, and encodes a lipase secreted in the pancreatic juice. MODY8 is a rare disease leading to pancreatic exocrine dysfunction that precedes beta cell alterations (54). Pelligrini et al. derived three iPS clones from the patient’s skin fibroblasts, and used them to generate beta cells by following the developmental stages. These beta cells were found to show normal insulin secretion in response to glucose. Thus, this study appears to be useful not only for *in vitro* modelling of the disease, but also for beta cell replacement studies.

## The lessons from the pancreatic organoids

Organoids are 3D *in vitro* culture systems generated from stem or progenitor cells (55) which can mimic the function of some organs, including the pancreas. Organoid models can be used to study the roles of genetic factors, as well as the microenvironment in T1 and T2D (56). For example, some organoids can recapitulate organ development, thereby allowing evaluation of the impact of developmental defects. However, one limitation of such analyses is that they often represent a developmental stage rather than a mature organ (57). More recently, the use of organoids to study MODY3 has gained interest. Truncation of HNF1a (p.Pro291ProfsX25) is the most common mutation associated with MODY3 (58); however, while impaired HNF1a signaling is known to play a role in its development, the exact molecular mechanism remained unidentified. To explore this question, Cujba et al. generated CRISPR/CAS9 engineered HNF1a^p.Pro291ProfsX25^ cells from hiPS, which they used to generate 3D organoids. Using this model, they found a reduction of the number of progenitors as well as reduced beta-cell differentiation. At the molecular level, HNF1a**^p.Pro291ProfsX25^** interacts with HNF1b and inhibits its function. In HNF1a**^p.Pro291ProfsX25^** hiPS derived organoids, overexpression of HNF1b increased the PDX1+ progenitors. Similarly, overexpression of HNF1b in the HNF1a**^p.Pro291ProfsX25^** hiPS cell line partially restored differentiation of the beta cells. Together, these data show that organoids can be used to model MODY3 and decipher the underlying intrinsic molecular mechanisms. To improve the representativeness of the human organoids, it is important to consider that T2D is a multi-organ metabolic disease, with a strong inter-relationship between organs. Interestingly, this is also the case for MODY 5, which displays a variable phenotype and age of onset, with interactions between several organs (66). Such consideration also has a strong impact on the pre-clinical steps of drug-therapy. To address this question, Tao et al. used a microfluidic multiorganoid system to reproduce the liver-islets axis (61). This technology allowed 30 days of 3D organoids co-culture under circulatory perfusion. A transcriptional analysis validated the activation of metabolically appropriate pathways. Moreover, under high glucose conditions, mitochondrial dysfunction and decreased glucose transport were detected both in the liver and organoid islets. Interestingly, this phenotype was rescued by metformin treatments. Thus, this new model has opened the door for the further investigation of multi-organ interaction and drug discovery.

## Drug discovery and the clinical trials

The use of patient derived iPS cells for beta cell replacement is currently under investigation at the clinical level (67). The first clinical trial conducted on iPS cells was initiated in 2014. In this trial, differentiated Retinal Pigment Epithelial Cells were transplanted into a patient in Japan without any safety concerns. However, one major limit was the discovery of a genomic mutation in the derived iPS cells (68). More recently, experimentation using VX-880 cells has shown that stem-derived islet cell therapy could be applied to achieve insulin independence among individuals with T1D (American Diabetes Association, News Release June 23-26 2023, Vertex Press Release Jan7, 2024 and clinical trial NCT04786262). Moreover, in a new trial (VX-264), the same VX-880 cells were encapsulated in a device designed to eliminate the need for immunosuppressants. This study remains ongoing in multiple centers and countries (NCT05791201). Thus, these trials strongly suggest that beta-cell replacement is feasible in human. The same strategy could further be used to treat MODYs.

Furthermore, as indicated previously, Pellegrini et al. generated iPS cells by reprogramming somatic cells derived from a MODY8 patient with recurrent episodes of hyperglycemia without obesity. Interestingly, the authors were able to generate iPS-MODY8-derived beta cells completely devoid of functional alterations (53). Of note, these beta cells were able to secrete insulin following glucose stimulation. These experiments raise the possibility of autologous cell replacement therapy for MODY8.

In addition to cell therapy strategy, the use of iPS cells for drug discovery has also been investigated. One target investigated in this manner is dual-specificity tyrosine regulated kinase 1A (DYRK1A), which is ubiquitously expressed and has been implicated in brain development and function. DYRK1A haploinsufficiency in mice has been shown to lead to severe glucose intolerance, reduced beta cell mass, and diabetes (69). However, other studies have indicated that inhibition of DYRK1A stimulates beta cell proliferation in humans (70, 71). Recently, Barzowska et al. used a human organoid model to demonstrate that a set of DYRK1A small molecules inhibitors can enhance beta cell proliferation and long-term insulin secretion, in addition to balancing glucagon levels (59).

Moreover, Ilegems et al. recently identified the HIF1a inhibitor PX-478 as a good candidate to improve the function of beta cells (60). They first hypothesized that the beta cell dysfunction in T2D results from metabolic hypoxic stress. They further showed that administration of a HIF1a inhibitor could improve beta cell function in db/db mice and streptozotocin induced diabetes models. They further validated these results in pancreatic human organoids exposed to high glucose treatments.

In conclusion, the newly developed models based on human iPS cells discussed herein have considerably contributed to increasing our knowledge on the molecular mechanisms underlying diabetes in humans. The use of organoids and beta cells derived from patient iPS cells have paved the way for advances in drug discovery and regenerative medicine which may ultimately allow treatment of diabetes patients with autologous beta cell replacement. Moreover, these models have helped to translate data identified in mouse models in human models, thereby increasing the robustness of preclinical data.
